# Identical repeated backbone of the human genome

**DOI:** 10.1186/1471-2164-11-60

**Published:** 2010-01-23

**Authors:** Cinthya J Zepeda-Mendoza, Tzitziki Lemus, Omar Yáñez, Delfino García, David Valle-García, Karla F Meza-Sosa, María Gutiérrez-Arcelus, Yamile Márquez-Ortiz, Rocío Domínguez-Vidaña, Claudia Gonzaga-Jauregui, Margarita Flores, Rafael Palacios

**Affiliations:** 1 Centro de Ciencias Genómicas, Universidad Nacional Autónoma de México, Cuernavaca, Morelos, 62210, México

## Abstract

**Background:**

Identical sequences with a minimal length of about 300 base pairs (bp) have been involved in the generation of various meiotic/mitotic genomic rearrangements through non-allelic homologous recombination (NAHR) events. Genomic disorders and structural variation, together with gene remodelling processes have been associated with many of these rearrangements. Based on these observations, we identified and integrated all the 100% identical repeats of at least 300 bp in the NCBI version 36.2 human genome reference assembly into non-overlapping regions, thus defining the Identical Repeated Backbone (IRB) of the reference human genome.

**Results:**

The IRB sequences are distributed all over the genome in 66,600 regions, which correspond to ~2% of the total NCBI human genome reference assembly. Important structural and functional elements such as common repeats, segmental duplications, and genes are contained in the IRB. About 80% of the IRB bp overlap with known copy-number variants (CNVs). By analyzing the genes embedded in the IRB, we were able to detect some identical genes not previously included in the Ensembl release 50 annotation of human genes. In addition, we found evidence of IRB gene copy-number polymorphisms in raw sequence reads of two diploid sequenced genomes.

**Conclusions:**

In general, the IRB offers new insight into the complex organization of the identical repeated sequences of the human genome. It provides an accurate map of potential NAHR sites which could be used in targeting the study of novel CNVs, predicting DNA copy-number variation in newly sequenced genomes, and improve genome annotation.

## Background

Approximately 45% of the human genome is composed of repetitive sequences including transposon-derived repeats, processed pseudogenes, simple sequence repeats, and blocks of tandemly repeated sequences [1], which we will refer to as common repeats. In addition to these elements, segmental duplications (SDs) constitute another kind of repeated sequences that compose around 5% of the genome. They have been defined as blocks of DNA that range in size from 1 to 400 kilobases (Kb), share a high level of sequence identity (>90%), and are present in at least two copies in the genome [2]. Both SDs and common repeats have been involved in non-allelic homologous recombination (NAHR) events, generating diverse genomic rearrangements [3-6].

NAHR is a major mechanism for the generation of genomic rearrangements during both mitosis and meiosis. For NAHR to occur there is a requirement of sequences sharing a high degree of identity with a minimal length of about 300 base pairs (bp) [7,8]. Besides size and sequence identity, genomic architectural features such as distance between repeats and orientation with respect to each other could influence recombination rates [3]. Genomic rearrangements have been associated with genomic disorders [9], and are major contributors to copy-number variation among humans. Copy-number variants (CNVs) are common in normal healthy individuals [10,11], and some of them appear to be related with gene dosage variation and disease susceptibility or resistance [12].

Based on the known principles for NAHR to occur, Sharp *et al. *predicted microdeletion and microduplication rearrangements between SDs in patients with idiopathic mental retardation [13]. In another study, Lam *et al*. analyzed the role of meiotic and mitotic recombination in the α-globin genes instability leading to deletions in blood and sperm cells [14]. In the same line, Flores *et al*. predicted and detected recurrent NAHR inversion rearrangements between inverted repeats with 100% identity and a size greater than 400 bp in somatic cells of normal individuals [15].

Given the importance of repeated sequences as players of continuous structural genome remodelling processes like the generation of genomic variation, occurrence of genomic disorders, and possible gene innovation [3,16], an analysis of these sequences is appropriate not only to identify potential substrates for NAHR events to occur, but also to gain insight into the current dynamic state of the human genome and its evolutionary past.

In the present work we identify and describe the nature of all the 100% identity repeated sequences of at least 300 bp in the public human genome reference assembly. Based on these data, we constructed the Identical Repeated Backbone (IRB). The IRB comprises around 2% of the total human genome and is localized across all human chromosomes in 66,600 non-overlapping regions. The IRB overlaps important structural and functional elements such as SDs, common repeats, and genes. In addition to providing a map for potential NAHR events, the IRB resource could be used to improve current database annotations, characterize new copy-number variable regions, and identify probable copy-number variable regions in newly sequenced genomes.

## Results

### Definition of the IRB

**Figure 1:**
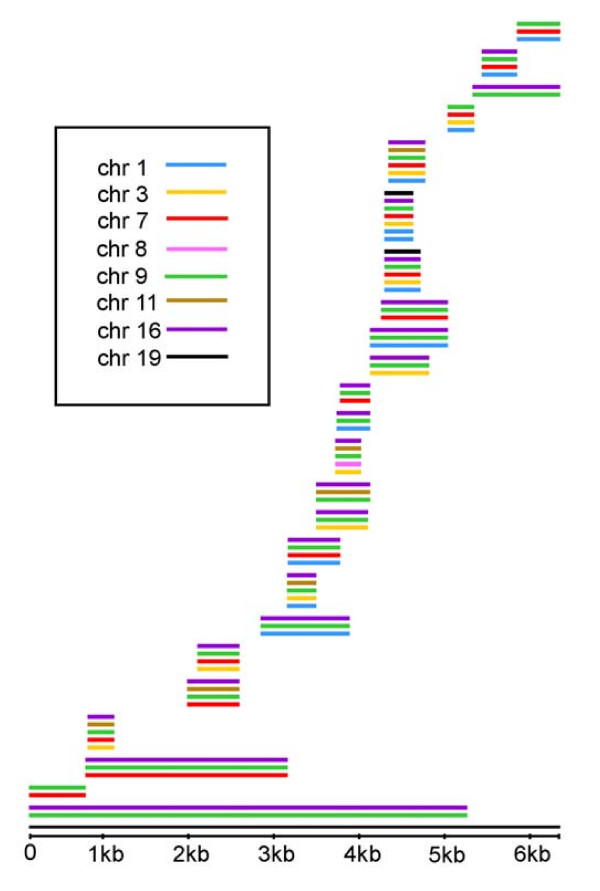
**General structure of complex ISTs**. An example of the ISTs that integrate the IRB is shown. Each line represents an IC and it is drawn according to its position on the IST. The black line at the bottom represents the IST sequence; the remaining colours represent the distinct chromosomes where the ICs that compose this IST are located.

For this study, the National Center for Biotechnology Information (NCBI) version 36.2 of the human genome reference assembly (hereafter NCBI assembly) was used. The IRB of the human genome reference assembly comprises every non-overlapping bp that is repeated in a context of at least 300 continuous identical bp. To construct the IRB, the NCBI assembly was first analyzed to find all the intrachromosomal and interchromosomal identical repeat pairs with a minimal length of 300 bp. We found 698,065 of such pairs. The members of each of these pairs are herein referred to as Identical Core sequences (ICs). Intrachromosomal paired ICs are separated by a median distance of 3 Megabases (Mb). However, 35% of the total pairs are located less than 1 Mb apart, and about half of these fall within a distance of less than 100 Kb (data not shown). ICs range in length from 300 to 88,815 bp with an average length of 448 bp (Additional file 1: table s1). Each IC is repeated from 1 up to 220 times, and they show in general a large degree of overlap among them. Overlapping ICs were then concatenated into larger non-overlapping sequence blocks, called Identical Sequence Tracks (ISTs). ISTs vary in complexity; simple ISTs are formed by a single IC while complex ISTs are constituted by two or more overlapping ICs (schematic representation of a complex IST is shown in Figure 1). The whole set of ISTs forms the IRB.

The IRB comprises 61,088,514 bp, which are equivalent to ~2% of the total NCBI assembly. It is localized throughout the genome in 66,600 ISTs regions that range in size from 300 to 130,815 bp with an average length of 917 bp (Additional file 1: table s1). The distance between ISTs varies from 1 to 30,000,252 bp with an average of 44,087 bp.

**Figure 2:**
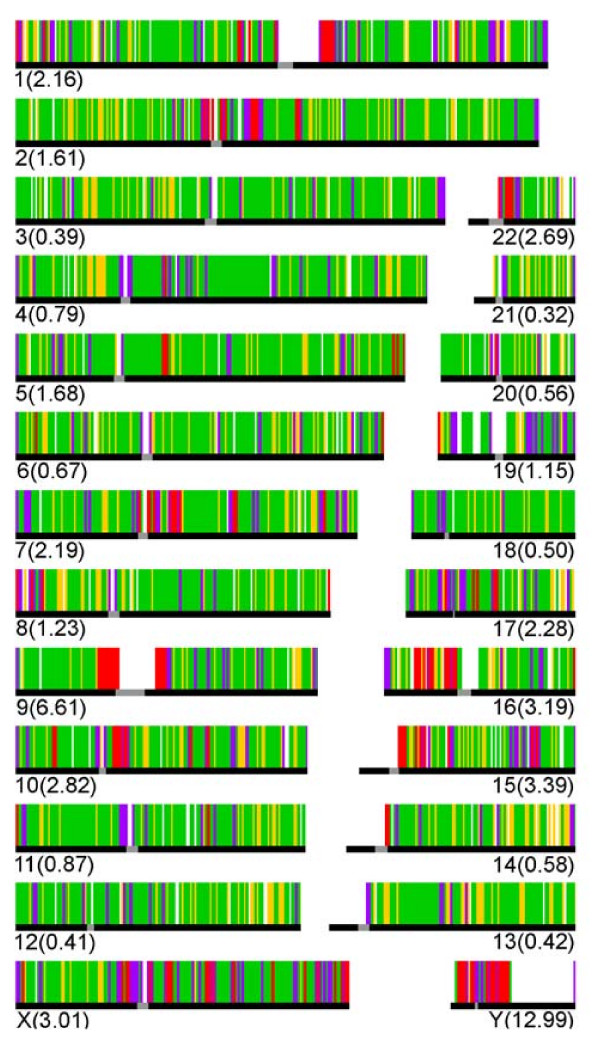
**IST densities of the human chromosomes**. The total genome was divided in 1 Mb windows and the total number of bp that belonged to ISTs within the window was counted. All the chromosomes are represented in the figure. The numbers between parentheses represent the percentage of the chromosome that pertains to the IRB. Yellow colour represents an IST density from 1 bp to <1 Kb per Mb; green, from 1 Kb to <10 Kb; purple, from 10 Kb to <100 Kb and red from 100 Kb to 1 Mb. Most white spaces represent gaps in the reference human genome.

When calculating the whole IRB bp percentage per chromosome, we found that the Y chromosome has the highest value (13). Among the autosomes, chromosome 9 and chromosome 21 have the highest (6.6) and lowest (0.3) IRB bp percentages, respectively. We also calculated what we called the repeated density of ISTs, which we defined as the number of bp belonging to ISTs in chromosomal windows of 1 Mb. As shown in Figure 2, the ISTs density varies widely across the genome. The lowest density found corresponds to 0 bp for 169 windows scattered throughout all chromosomes, while the highest corresponds to a region on the Y chromosome with a density of 994,635 ISTs bp per Mb. The highest ISTs density regions are frequently located near centromeres and telomeres.

It is important to mention that the pseudoautosomal regions shared by chromosomes X and Y were not considered as repeated sequences in this analysis (see Methods). The complete list of ICs and ISTs are publicly available and can be accessed at http://paris.ccg.unam.mx/hsapiens/IRB/RepeatCoreJoining.txt and http://paris.ccg.unam.mx/hsapiens/IRB/CoreAllTracks.txt, respectively.

### Analysis of Common Repeats and SDs in the IRB

A major feature of the genomes of higher organisms is the presence of diverse types of highly reiterated elements. It has been reported that common repeats comprise about 45% of the reference human genome [1]. We used Repeat Masker to identify the different types of common repeats in both the IRB and the NCBI assembly. We found that 54% of the IRB (33,199,901 bp) corresponds to these elements, in contrast to 45.4% (1,399,601,346 bp) detected in the whole human genome.

A comparison of the common repeat types detected in the IRB and in the total genome is shown in Table 1. Notably, the ratio of LINEs over SINEs is higher in the IRB (2.1) than when considering the total genome (1.5). There is also an enrichment of satellite type DNA (4.9) and an underrepresented proportion of DNA transposons (3.7) when compared to the complete reference sequence (0.8 for the satellite and 6.8 for the DNA transposons, respectively).

**Table 1:** Masked bp in total genome and IRB

Type of element	Bp in total genome (a)	Bp in IRB (a)
LINE	602817717 (43.1)	15825138 (47.7)
SINE	391097725 (27.9)	7358371 (22.2)
LTR	253911472 (18.1)	5707753 (17.2)
RNA	1082672 (0.1)	39790 (0.1)
Satellite	11217284 (0.8)	1614833 (4.9)
DNA transposons	94488985 (6.8)	1237710 (3.7)
Low Complexity	40301995 (2.9)	962052 (2.9)
Unknown	4683496 (0.3)	454254 (1.4)
**Total**	**1399601346 (100)**	**33199901 (100)**

**a = % of masked sequence**. A comparison of the ratios of the major repeated elements in the IRB and the reference human genome is shown in parenthesis.

Following the analysis of known repeated sequences within the IRB, we performed a comparison of the IRB against the catalogue of SDs from the Human Genome Segmental Duplications Database of March 2006 [17]. We found that about 80% of the IRB overlaps with SDs. Due to our 100% identity analysis parameter, approximately 66% of these IRB bp overlap with more than 99% identity SDs. Accordingly, all the bp of SDs reported as identical fall within the IRB http://paris.ccg.unam.mx/hsapiens/IRB/IRB_SDs_comparisons.txt. Although reported as SDs [17], in this study we did not consider the pseudoautosomal regions of the X and Y chromosomes as duplicated regions (see Methods).

### Genes in the IRB

To search for genes within the IRB, we compared the Ensembl release 50 annotation of human genes [18] to the ICs used to construct the IRB (see above). It is important to remind that, given that the ICs were identified in pairs, each gene contained within the ICs should be part of a set with at least two copies with 100% identity.

**Figure 3:**
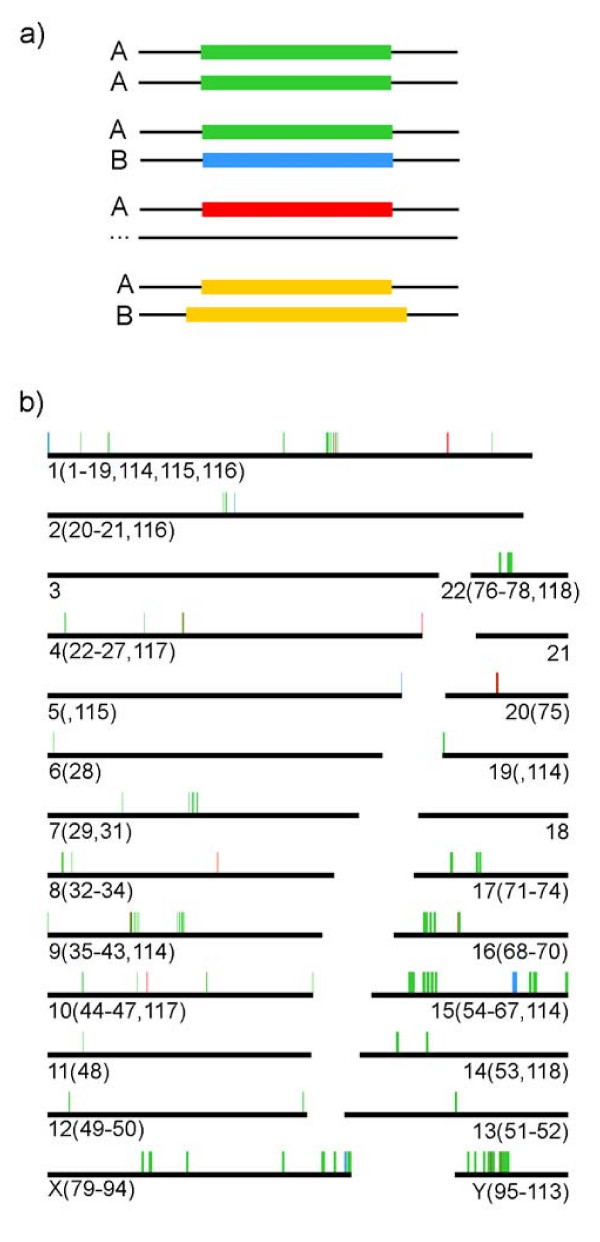
**Genes in the IRB**. A) Scheme of the distinct cases that were found in analyzing the nature of the genes. Green colour means that all gene data are congruent between the set and Ensembl, blue colour represents function incongruence, red colour represents that at least one of the gene copies is not annotated in the Ensembl database, yellow colour means incongruence in size. B) Genes that were totally comprised by the IRB are shown in their respective chromosomes. Each bar represents a gene. The numbers between parentheses represent the sets of genes that are present in each chromosome; numbers that follow the comas are the names of the sets that have copies of their elements in other chromosomes.

The complete Ensembl list was filtered to include protein coding genes and different types of non-coding RNA genes, and to exclude pseudogenes, leaving a total of 26,771 elements. We found 268 Ensembl genes contained within the ICs. We clustered the genes and inferred the presence of 118 sets (Additional file 1: table s2). Most of the sets comprise 2 elements; however, 26 sets include 3 to 14 genes. We detected four different categories of gene sets in regard to the congruence between the Ensembl annotation and the IRB: a) complete consistency, all the elements in the set coincide in the size, position and functional description reported by Ensembl; b) size inconsistency, all the elements in the set are reported in Ensembl with the same description, but the reported length of at least one member is different. In some sets, one of the reported elements extends beyond the 100% identity boundaries; c) description inconsistency, at least one member of the set is annotated as a pseudogene within the boundaries of the corresponding IC; d) absence in Ensembl annotation, at least one of the members of the set is not reported in Ensembl (Figure 3A).

Most of the sets, 91 out of 118, belonged to the group showing complete consistency, 5 sets showed size inconsistency, 6 sets presented description inconsistencies, and in 12 sets at least one gene was not annotated in Ensembl. A total of 15 genes were not present in the Ensembl database. Of these, three are GOR antigen protein fragments, two correspond to fragments of D4S2463 homeobox-like proteins, one is a TP53-target gene 3 protein (*TP53TG3*), one is a double homeobox protein 4 (*DUX4*), one is a 93 bp novel miRNA predicted from RFAM and miRBase, and seven are identical to genes annotated as uncharacterized proteins. The locations of the genes without annotation in Ensembl were inferred from the positions of the annotated elements in the corresponding ICs. To ascertain the existence and the accuracy of the positions of the proposed identical elements, we obtained the sequences of the predicted genes from the NCBI assembly and performed global alignments among all the members of the corresponding gene set. As expected, all the alignments showed 100% identity.

When the sets presented size inconsistency within the ICs (Figure 3A), the positions of the shortest element were adjusted to those of the largest element and global alignments were performed. In all cases 100% identity with the largest element was found. The sets of group c, where a pseudogene was annotated, also presented size inconsistency. In these cases we adjusted the positions of the corresponding region to those of the annotated gene in the set. Global alignments revealed a 100% sequence identity among the proposed and the previously annotated genes. We found a particular case in which a set (set number 70, Additional file 1: table s2) presented three elements: two genes annotated as *TP53TG3*, with an inconsistency in their reported sizes (4,353 and 5,777 bp), and a non-annotated gene with a size of 4,353 bp. In this case it is difficult to ascertain the actual size of the gene.

It should be noticed that the number of identical genes presented here could be an underestimation, since some of them could be present in regions that do not meet the criteria of at least 300 identical bp to be considered as ICs (see above). In order to know the actual number of identical gene copies, each gene detected in the IRB was aligned against the NCBI assembly. The analysis revealed one extra identical gene copy for sets 34, 42, 66, and three for the sets 1 and 53. In addition, in two cases genes from different sets revealed to be identical (sets 1-53 and 18-19). This was due to the fact that although the genes were identical, the 300 bp regions needed to be considered as IC pairs were different. As a result, these genes were classified into different IC-gene sets. The identical genes found in this analysis were finally associated in a total of 116 groups of identical genes (Additional file 1: table s2).

Overall, including the previously non- and miss-annotated genes, we found 296 genes in the IRB, distributed in 116 groups. The largest group of genes comprises 16 copies of a novel 119 bp rRNA gene predicted from RFAM and miRBase. Of the 296 genes, there are 145 non-coding RNAs of different types: 26 rRNA; 42 snRNA; 10 snoRNAs; 39 miRNAs; and 27 described as miscellaneous RNAs. It is interesting to highlight that the ratio of rRNA, snRNA, miscRNA and miRNA genes over the total IRB genes compared to their ratio in the Ensembl database, is significantly higher (7, 3, 2.6 and 2.5 respectively) (Additional file 1: table s3). The other 151 elements correspond to protein coding genes; of these, 93 genes have an assigned function, and in 29 of them an expression in testis has been observed. Actually, 22 of these genes encode cancer-testis antigens localized in the X chromosome. Finally, 58 genes did not have an assigned function, and no retrotransposed or scRNA genes were detected.

There is an overall bias towards small genes in the IRB as expected from the size and high identity thresholds used for the analysis. For non-coding RNA genes, the average length is 118 bp. For protein genes, the average size is 3,379 bp compared to the average 100 kb size for the whole genome [1]. Actually, only 6 genes of the IRB are larger than 10 Kb. The two largest identical genes have a size of 45,273 bp, and correspond to protein coding genes without an assigned function located in chromosome 9. These genes contain 15 exons with a total length of 1,881 bp and 14 introns corresponding to 43,392 bp. Intrachromosomal identical repeated genes compose the majority of the sets, while only 5 sets comprise interchromosomal members.

### CNVs in the IRB

In 2004, Sebat *et al. *and Iafrate *et al. *papers were the first ones to indicate a widespread distribution of genomic copy-number variable regions among healthy individuals [10,11]. Since then, hundreds of new CNVs have been described in the human genome thanks to the use of comparative genome analysis and array technologies. Depending on their genomic location, these structural variants may alter gene dosage, gene expression or gene function [reviewed in [16,19,20]]. When we compared our IRB against regions already identified as copy-number variable in the Database of Genomic Variants (DGV) [21], we encountered that about 73% of our ISTs overlapped regions catalogued as CNVs. This corresponds to ~81% (49,377,648 bp) of the total IRB bp, and around 16% of the total CNVs bp. It is worth mentioning that 99.7% of the overlapping ISTs were completely embedded within the CNV regions. Expanding the analysis of these regions, we found that ~89% of the total IRB bp which overlap CNVs are catalogued as SDs.

In addition to the aforementioned examination, we performed a comparative analysis of the IRB against detected CNVs in the Watson [22] and Venter genomes [23] (see Methods). We found that 1,148 (1,055,668 bp) and 563 (641,194 bp) regions of the IRB overlapped with CNVs in the Watson and Venter genomes, respectively. A comparison involving the CNV regions in the DGV and the Watson and Venter genomes against the IRB revealed a shared number of 89 CNVs (42,568 bp) which overlap the IRB sequence.

### Evidence for gene copy-number variation in the IRB in two diploid human genomes

Given that the IRB sequences fulfill the minimal requirements of length and identity to promote recombination events, we can expect to find evidence of IRB-driven rearrangements that may involve functional elements such as genes. Copy-number variable genes might have a higher or lower number of reads spanning these regions, which could indicate possible gene duplications or deletions, respectively. To test this hypothesis, we performed an "*in silico *hybridization" of 52 non-coding RNA genes within the IRB with a size ranging from 82 to 167 bp, and compared them against the raw sequence reads of the Venter and Watson genomes. We restricted the analysis to short RNA genes due to the average lengths of the Watson genome reads (250 bp), therefore reducing the noise on the BLAST searches to obtain *bona fide *hits [22].

The sequence of each of the 52 non-coding RNAs was used as an *in silico *BLAST probe against the 74,198,831 and 31,861,638 reads of the Watson and Venter genomes, respectively. The total number of 100% identity hits is summarized in Table 2. To assess the significance of the results we compared the RNA genes hit number with the hit number of 220 randomly chosen fragments of the average size of the RNA genes from the NCBI assembly that produced a single hit in the reference genome. The random fragments had, on average, 2.5 hits (sd = 2) in Watson's genome and 5.2 (sd = 2.7) hits in Venter's genome, respectively. The ratio of each of the RNA gene hits in either Watson or Venter over the reference assembly was compared against the random fragments statistics. Using a threshold of 2 standard deviations, most of the RNA genes analyzed had a representation close to the average of the random fragments, suggesting a proportional copy number with the reference human genome. However, two novel *5S_rRNA *genes in the Venter genome, and one novel misc_RNA gene and two novel *5S_rRNA *genes in the Watson genome presented a higher than average hit ratio in both diploid genomes when compared to the NCBI assembly. On the other hand, six of the studied genes had no 100% identity hits in the Watson or Venter genomes (one novel rRNA, a snoRNA, a novel miscRNA, and four novel snRNAs) (see Table 2). Unlike the cases of significant gene duplications, the deletions results should be interpreted with caution (see Discussion).

**Table 2:** *In silico *hybridization of IRB genes against Venter and Watson genomes

						Hit number			Ratios	
	#	Gene name	Type	Ensembl ID	Size	Ref	Vent	Wat	Vent/Ref	Wat/Ref
	1	AC006328.5	Novel miRNA	ENSG00000221640	82	2	8	2	4.0	1.0
	2	AC116165.7	Novel miRNA	ENSG00000221000	82	4	17	10	4.3	2.5
	3	AC123768.8	Novel miRNA	ENSG00000221405	82	2	10	7	5.0	3.5
	4	hsa-mir-1233	Known miRNA	ENSG00000221065	82	2	5	2	2.5	1.0
	5	AC119751.3	Novel miRNA	ENSG00000221212	83	2	9	2	4.5	1.0
	6	AC135995.7	Novel miRNA	ENSG00000221095	83	3	3	3	1.0	1.0
	7	AC136698.6	Novel miRNA	ENSG00000221008	83	3	20	4	6.7	1.3
	8	SCARNA18	Novel misc_RNA	ENSG00000212253	83	3	13	14	4.3	4.7
	9	hsa-mir-511-2	Known miRNA	ENSG00000207937	87	2	6	2	3.0	1.0
	10	hsa-mir-514-3	Known miRNA	ENSG00000207866	88	2	5	6	2.5	3.0
	11	SNORD103	Known snoRNA	ENSG00000200154	91	2	17	9	8.5	4.5
	12	AC147055.2	Novel miRNA	ENSG00000212033	93	2	6	1	3.0	0.5
	13	AC068704.4	Novel miRNA	ENSG00000221682	95	2	10	1	5.0	0.5
	14	Y_RNA	Novel misc_RNA	ENSG00000206706	98	4	19	10	4.8	2.5
	15	U6	Novel snRNA	ENSG00000201789	99	2	15	3	7.5	1.5
	16	hsa-mir-1184	Known miRNA	ENSG00000221190	99	3	6	3	2.0	1.0
	17	Y_RNA	Known misc_RNA	ENSG00000201138	100	2	6	7	3.0	3.5
	18	Y_RNA	Novel misc_RNA	ENSG00000199641	100	9	55	26	6.1	2.9
	19	AC137056.3	Novel mi_RNA	ENSG00000221119	102	2	10	6	5.0	3.0
^1^	20	AC019322.8	Novel misc_RNA	ENSG00000200514	103	2	13	18	6.5	9.0
	21	U6	Novel snRNA	ENSG00000206655	103	2	3	1	1.5	0.5
	22	AC068020.7	Novel miRNA	ENSG00000221027	104	2	13	3	6.5	1.5
	23	AL031963.40	Novel miRNA	ENSG00000221162	105	2	10	3	5.0	1.5
	24	U6	Novel snRNA	ENSG00000212612	107	3	7	4	2.3	1.3
	25	U6	Novel snRNA	ENSG00000212419	107	4	22	15	5.5	3.8
	26	U6	Novel snRNA	ENSG00000206804	107	2	17	6	8.5	3.0
	27	U6	Novel snRNA	ENSG00000206972	107	2	8	8	4.0	4.0
	28	U6	Novel snRNA	ENSG00000200493	107	3	14	6	4.7	2.0
	29	BX842679.19	Novel rRNA	ENSG00000191555	108	2	4	8	2.0	4.0
	30	AL627230.15	Novel misc_RNA	ENSG00000199432	110	4	22	6	5.5	1.5
	31	5S_rRNA	Novel rRNA	ENSG00000212154	112	2	2	4	1.0	2.0
	32	5S_rRNA	Novel rRNA	ENSG00000212173	113	2	9	2	4.5	1.0
^2^	33	5S_rRNA	Novel rRNA	ENSG00000206584	116	2	4	0	2.0	0.0
	34	5S_rRNA	Novel rRNA	ENSG00000200336	118	2	11	5	5.5	2.5
^1^	35	5S_rRNA	Novel rRNA	ENSG00000199270	119	16	216	162	13.5	10.1
^1^	36	5S_rRNA	Novel rRNA	ENSG00000201925	119	16	216	162	13.5	10.1
	37	SNORA11D	Known snoRNA	ENSG00000221475	128	2	5	3	2.5	1.5
	38	AC019322.8	Novel snoRNA	ENSG00000206793	133	2	4	2	2.0	1.0
	39	hsa-mir-1302-2	Known miRNA	ENSG00000221661	138	4	17	10	4.3	2.5
^2^	40	AC006983.4	Novel snoRNA	ENSG00000207143	139	2	6	0	3.0	0.0
	41	SCARNA17	Novel misc_RNA	ENSG00000212286	143	2	8	2	4.0	1.0
^2^	42	U1	Novel snRNA	ENSG00000207519	154	2	1	0	0.5	0.0
	43	U1	Novel snRNA	ENSG00000206945	160	2	6	1	3.0	0.5
	44	U1	Novel snRNA	ENSG00000202064	161	2	9	4	4.5	2.0
	45	U1	Novel snRNA	ENSG00000207273	162	2	9	3	4.5	1.5
^2^	46	U1	Novel snRNA	ENSG00000201183	162	2	3	0	1.5	0.0
	47	U1	Known snRNA	ENSG00000207389	164	7	62	15	8.9	2.1
	48	U1	Novel snRNA	ENSG00000207226	164	2	5	1	2.5	0.5
^2^	49	U1	Novel snRNA	ENSG00000206585	164	2	3	0	1.5	0.0
	50	U1	Known snRNA	ENSG00000206588	164	7	62	15	8.9	2.1
^2^	51	U1	Novel snRNA	ENSG00000206828	164	2	0	3	0.0	1.5
	52	U1	Novel snRNA	ENSG00000201105	167	2	6	4	3.0	2.0

52 X non-coding RNA genes within the IRB were compared against the raw sequence reads of the Venter and Watson genomes.

^1 ^- Genes whose copy number is higher when compared to the NCBI assembly.

^2 ^- Genes who had no hits on the Venter or Watson genomes.

## Discussion

In this study we identify, consolidate, and analyze all the 100% identity repeated sequences in the human genome that have a length of at least 300 bp. The result of our analysis, the IRB, comprises around 2% of the total reference human genome, and includes potential recombinogenic sites which overlap important functional and structural elements such as SDs, common repeats, and genes.

Because almost half of the total bp in the human genome (45%) corresponds to common repeats, it is not surprising that common repeats comprise 54% of the IRB bp. We observed an enrichment of LINEs over SINEs in the IRB compared to the total genome, as well as an underrepresented proportion of DNA transposable elements versus the total genome. These biases could be explained by size differences among these elements. SINEs have a length of about 100-400 bp, and the average size for all LINE1 copies (the most abundant LINE elements) is 900 bp (overall, LINEs are about 6-8 Kb long) [24]. In the same way, DNA transposon fossils range from 2-3 Kb for the autonomous type and from 80-3000 bp for non-autonomous type [25]. As in our analysis we look for 100% identity repeats of at least 300 bp, this length range may reduce the number of expected versus found rates of SINEs compared to LINE elements, and of DNA transposons compared to the total genome ratios. Another explanation for the overabundance of LINEs over SINEs and underrepresentation of DNA transposons might be related to the percentage identity threshold, as a single mismatch might break the length of identical sequences lower than the detection minimum of 300 bp, thus making the used algorithms overlook the regions. Therefore, SINEs, which have a shorter average size compared to LINEs, could be underrepresented in the IRB due to slight variations in their sequence; DNA transposons could be subject to the same explanation.

An enrichment of satellite type DNA was also detected in our dataset. Satellite DNA is known to be present in several centromeric and pericentromeric regions in the human genome [26]. For example, alpha satellite DNA is found on all human chromosomes, while beta satellite DNA is normally present in tandem arrays of acrocentric chromosomes, covering hundreds of Kb. In addition, telomeric DNA accounts for many Kb located at the termini of human chromosomes. Even though mutations can exist in satellite sequences, long stretches of satellite type DNA conserve the established 100% identity and 300 bp thresholds. As a result, more satellite type DNA elements would be included in the IRB, explaining the observed enrichment.

SDs are another interesting feature of the human genome. Because SDs are large, highly identical sequences interspersed throughout the genome, it is expected that most of the IRB bp fall within this classification. In fact, we observed that 80% of the IRB overlapped with SDs, with 66% of the ISTs overlapping SDs of >99% identity. Correspondingly, ~33% of the total SD bp overlap with the IRB bp. These numbers reinforce the general idea that the IRB contains potentially recombinogenic sites, as SDs are known substrates of homologous recombination events [3,4].

A major result of our analysis is concerned with the presence of genes in the IRB. We found 296 genes which are completely contained within ICs. Of the 296 genes, 145 are classified as non-coding RNAs. Of these, approximately one third are annotated as miRNAs, accounting for ~3% of all human miRNAs. This is an interesting result because it has been observed that miRNAs play important roles in many biological processes such as cell growth and differentiation, apoptosis, and gene regulation [27]. In this sense, it could be possible to correlate and/or make predictions of potential disease phenotypes based on the knowledge that these genes are prone to rearrangements. Actually, it has been reported that frequent deletions of miRNA genes *miR15 *and *miR16 *occur on patients with chronic lymphocytic leukaemia, suggesting a possible role for these miRNAs in the generation of this type of cancer [28]. On the other hand, given that miRNAs function as fine-tuners of gene expression, it would be interesting to analyze the role of genomic rearrangements that include miRNAs throughout evolution.

Of the 296 identical genes identified, we found elements of the Golgin subfamily A and the Double homeobox family which, when compared to the Rhesus macaque genome, were described as gene families with a significant copy-number expansion in human [29]. Another interesting observation is that an expression in testis has been reported for one third of the protein-coding genes detected in the IRB. Of these genes, 92% (22 genes) are members of the cancer-testis antigen family located in the X chromosome. It is known that most of the cancer-testis genes located in this chromosome are members of families that fall within complex regions of direct and inverted repeats, and have been reported to be undergoing expansion through duplication events [30].

We are aware that the number of genes detected in the IRB could be an underestimation of the total identical genes in the human genome, since they may not meet the length threshold that we used for this analysis. In spite of this, the utility of using identical sequences enabled us to notice 26 inconsistent cases in the Ensembl database v50 human genome annotation. These include 5 identical genes with different annotated sizes but with the same description, 6 identical genes with different annotated descriptions, and 15 regions identical to a gene but not annotated as such. By considering the stringent identity threshold used to construct the IRB, the IRB-based gene analysis could be used as a suitable tool for refining annotation details of many different databases.

It is important to notice that the IRB includes identical pairs of long sequences, up to 88 Kb. The fact that no SNPs or indels were found is indeed odd, but these data are based directly in the reported sequence of the reference human genome. Until now, the reference assembly has the highest degree of accuracy available for any sequenced organism, with a calculated error rate of 1/100,000 bp [31]. Any sequence or assembly errors in the reference would be translated into errors in the actual IRB, however this is not ascertainable *in silico*. Nonetheless, another plausible explanation for the high identity of the regions within the IRB is that they might have been duplicated recently in evolution; it could also be possible that they are undergoing frequent gene conversion. These regions might also be polymorphic within the human population. We encountered that around 73% of our ISTs overlapped CNV regions from the DGV, which correspond to 81% of the total IRB bp. It is worth noticing that almost all of the overlapping ISTs were completely included within the CNV regions. An important observation is the fact that ~89% of the overlapping ISTs-CNVs sequence is catalogued as SDs. Previous studies have reported a significant association of CNVs with SDs [32], which might suggest a SD-mediated mechanism for the generation of these CNVs.

Most interesting to notice is the degree of overlap that exists between the IRB and CNVs detected in other sequenced genomes. A comparison of the identified CNV regions in the Watson and Venter genomes revealed an overlap of 1,055,668 and 641,194 bp with the IRB, respectively. Moreover, a comparison among the DGV, and the Watson and Venter CNVs, brought to the fore shared regions of copy-number changes that overlap the IRB. Overall, these observations suggest that the ISTs might be participating as substrates for recombination events, which might ultimately lead to genomic rearrangements and copy-number changes. Following this hypothesis, we might expect to find CNV regions associated with the remaining 17,932 (27%) ISTs, which might not have been yet identified as CNVs, either due to technical limitations of current methods or lack of populations sampling.

Expanding the CNVs-gene analysis of the IRB, we searched for possible gene copy-number variations in the Watson and Venter genomes by comparing 52 non-coding RNAs against the NCBI assembly. By using pair-wise alignments it was found that most of the genes analyzed had at least one identical hit in the three genomes, and most of them had a hit number close to the average of a control set of randomly chosen small fragments of the reference assembly. We found statistically significant duplication evidence for two genes in the Venter genome (two cases of novel *5S_rRNA*) and three genes for the Watson genome (a novel misc_RNA, and two novel *5S_rRNA *genes). We also had six cases where no hits were detected in the diploid genomes (five genes for Watson and one for Venter). These genes include a novel *5S_RNA*, a novel misc_RNA, and four novel copies of the *U1 *gene. We must be careful when interpreting these zones as possible deletions in the Venter or Watson genomes, mainly because the absence of hits for these genes could have been produced by sequencing errors, different coverage of the genomic regions, or by sequence polymorphisms (single nucleotide polymorphisms (SNPs), insertions and deletions). Additional analysis revealed that of the 9 cases of genes which presented no hits or a higher number of hits, none overlapped any Watson or Venter reported CNVs (data not shown).

It is tempting to speculate that the identified copy-number variable genes for the Watson and Venter genomic regions could be possible *de novo *duplications/deletions for either individuals, or deletions/duplications for the reference assembly. However, it is important to highlight that significant local fluctuation in read depth across the Venter and Watson genomes and the NCBI assembly, together with the presence of SNPs and microindels, might limit the ability for an accurate *in silico *CNV prediction with our methodology. Nevertheless, the possibility of identifying novel CNVs for two recently sequenced genomes is a step towards the discovery of other new copy-number variable regions in the human genome. Further experiments must be performed to verify if the predicted regions are true CNVs.

As a final remark, it is important to consider that the IRB need not to be identical in different individuals due to the presence of SNPs, microindels, and structural variation. Comparisons among the IRB of the reference human genome and recently published personal genomes will be plausible once newly sequenced genomes attain a higher degree of assembly confidence to make appropriate definitions of their individual IRBs. For now, IRB and raw sequence reads comparisons have shed light on important functional and structural aspects of the identical repeated nature of the reference human genome assembly in regard to two other sequenced genomes. Furthermore, given that most of the resequenced personal genomes rely greatly on mapping sequence reads back to the reference assembly, the IRB of the reference assembly will also help to pinpoint highly identical regions in the new genomes.

## Conclusions

We have developed a framework for the study of repeated sequences that can be useful in analyses of genome structure and dynamics. By providing a map of potential sites for NAHR events to occur, the IRB is a new dataset which could help to target and identify novel copy-number variation and structural changes that might have functional implications. In this regard, the IRB is a good approach to start understanding the potential genotype-phenotype relationships of regions subject to copy-number changes. The analysis of these zones could also be coupled to the use of raw sequence reads from newly sequenced genomes to make *in silico *predictions of new CNVs. In addition, the methodology used to derive and analyze the IRB might also be a useful tool for improving some genome annotation inconsistencies.

The IRB analysis can be applied to many other sequenced organisms to help us understand the changes that genomes have undergone through their evolutionary road, and to elucidate the processes that have shaped their structures. In this particular case, through the analysis of the IRB we could expand our appreciation of the unique and complex repetitive nature of the human genome from a different point of view.

## Methods

### Detection of identical repeated sequences

Intrachromosomal and interchromosomal identical repeat pairs with a length of at least 300 bp in either direct or inverse orientations were identified in the human genome reference assembly of the NCBI build 36.2 using the programs Reputer version 3.0 and MUMmer version 3.19. The pseudoautosomal-regions of the X and Y chromosomes were excluded from the analysis because these sequences were not derived from a duplication event during evolution. Rather, their high degree of identity is the actual observation of a pair of chromosomes that diverged but conserved parts of their ancestral sequence.

Since one repeat can be part of multiple pairs, all couples were separated into their component repeats to generate a non-redundant list whose members are named Identical Core sequences (ICs). Each IC possesses a unique identifier and belongs to an IC family. Members of the same family have the same sequence although they are located in different chromosomal positions; by the use of families we are able to recover all original repeat pairs in the genome, which were later used in the gene analysis.

### IRB assembly

In order to obtain a non-redundant positions list for all the ICs, overlapping ICs were iteratively concatenated into larger sequence blocks. For example, consider ICs A, B and C. If A and B share a segment of their chromosomal positions, then they were fused into a longer element. If IC C did not overlap with any other IC, then its chromosomal position was unaltered. After this process the sets of the fused and unaltered ICs were called Identical Sequence Tracks (ISTs). As a result, ISTs can be formed by one or more ICs, with either intrachromosomal or interchromosomal classification, and direct or inverse orientation. The overall set of ISTs is considered to be the Identical Repeated Backbone (IRB).

### Density Measurements

In order to obtain the density of the ISTs in the human genome we divided the NCBI assembly in windows of 1 Mb and counted the total number of bp that belonged to IRB using custom-made Perl scripts.

### Finding common repeats

To identify all common repeats present in the IRB, the ISTs were masked for all types of interspersed repeats and low complexity DNA sequences using the default parameters as described in A.F.A. Smit, R. Hubley and P. Green RepeatMasker version 3.1.8 at http://repeatmasker.org. Results were analyzed with custom-made Perl scripts developed by our team.

### Presence of SDs

Overlap analyses were performed comparing the IRB to the SD list from the Human Genome SDs Database of March 2006 (Build36) http://humanparalogy.gs.washington.edu. To identify the number of bp in the IRB that overlap SDs regardless of their identity, overlapping SDs were concatenated to obtain a non-redundant list. We then separated the original SD list by their identity percentage, considering indels, and compared each set with the ISTs. Data were obtained and analyzed with custom-made Perl scripts developed by our team.

### Finding genes in the repeated sequences

We downloaded the list of human annotated genes from the Ensembl database http://www.ensembl.org/index.html release 50 without any filters, retrieving a total of 36,396 genes. Only 26,771 genes with an Ensembl biotype of protein-coding, miRNA, misc_RNA, retrotransposed, rRNA, scRNA, snoRNA and snRNA were considered for the analysis. We compared the reported positions of each gene with each of the original ICs that form the IRB with a custom-made Perl script. We focused on those genes that were totally included in the IRB.

### CNVs comparison

We downloaded the hg18.v7.mar.2009 version of the Database of Genomic Variants http://projects.tcag.ca/variation/ and excluded the CNVs with Levy or Wheeler references (to use for later comparisons with Watson and Venter CNVs). CNVs positions for the Watson and Venter genomes were obtained from the supplementary material provided by the corresponding articles. We used custom-made Perl scripts to compare the positions of each CNV with the IRB.

### James Watson and Craig Venter genomes comparisons

The raw sequence reads from the Watson and Venter genomes were downloaded from the NCBI ftp site ftp://ftp.ncbi.nih.gov/genomes/H_sapiens/. We used the genes mentioned in Table 2 as probes to detect copy-number variations. We first compared these genes against the human reference genome sequence and the Watson and Venter sequence reads, using WU-BLAST 2.2.6 with the default parameters. As a statistical control for assessing copy-number variations in the three genomes, we compared the gene results with the hybridization numbers of 220 randomly chosen fragments of X length. Only hits with 100% identity were considered.

## Authors' contributions

CZ, OY and RP conceived the study and outlined data analysis tasks. CZ, TL, OY, DG, DVG, KFMS, MGA, YMO, RDV, and CGJ performed data acquisition and filtering. CZ, TL and OY performed data analysis with help from DG. RP, MF, CGJ, and RDV provided feedback on results obtained. CZ, TL, OY and RP drafted the manuscript together. All authors read and approved the final manuscript.

## Authors' information

CZ, TL, OY, DVG, KFMS, MGA, YMO, RDV, and CGJ were undergraduate students at the time the IRB study was performed. At the time of manuscript submission CZ, TL, OY, DVG, KFMS, YMO, RDV, and CGJ hold graduate student positions at the following institutions:

**CZ**.- Watson School of Biological Sciences. Cold Spring Harbor Laboratory, 1 Bungtown Road, Cold Spring Harbor, NY 11724, USA.

**TL.- **Genome Sciences Graduate Program. University of Washington. Foege Building S-250, 1705 NE Pacific St, Seattle, WA 98195-5065. USA.

**OY**.- IMP - Research Institute of Molecular Pathology, Dr. Bohr-Gasse 7, 1030 Vienna, Austria.

**DVG**.- Instituto de Fisiología Celular, UNAM. Apartado Postal 70-600. Circuito Exterior s/n, Ciudad Universitaria, Delegación Coyoacán, CP 04510, México, D.F.

**KFMS**.- Instituto de Biotecnología, UNAM, Av. Universidad s/n, Col. Chamilpa, CP 62210, Cuernavaca, Mor. México.

**YMO**.- Max F. Perutz Laboratories, Medical University of Vienna, Dr. Bohrgasse 9/3, A-1030, Vienna, Austria.

**RDV**.- Interdepartmental Program in Cellular and Molecular Biology, Baylor College of Medicine, Houston, Texas, USA.

**CGJ**.- Department of Molecular and Human Genetics, Baylor College of Medicine, Houston, Texas, USA.

## Supplementary Material

Additional file 1 **Supplementary tables S1, S2, S3 **Excel file that contains ICs descriptive statistics, the positions of all the genes that were completely contained within the IRB and the IRB positions they overlap, and IRB-NCBI reference genome gene ratios. Click here for file
